# Preparation and Characterization of Perforated SERS Active Array for Particle Trapping and Sensitive Molecular Analysis

**DOI:** 10.3390/bios9030093

**Published:** 2019-07-25

**Authors:** István Rigó, Miklós Veres, Tamás Váczi, Eszter Holczer, Orsolya Hakkel, András Deák, Péter Fürjes

**Affiliations:** 1 Institute for Solid State Physics and Optics, Wigner Research Centre for Physics, Konkoly-Thege Miklós út 29–33., HAS, 1121 Budapest, Hungary; 2 Institute of Technical Physics and Materials Science, Centre for Energy Research, Konkoly-Thege Miklós út 29–33., HAS, 1121 Budapest, Hungary

**Keywords:** surface enhanced Raman spectroscopy, SERS array, bulk micromachining, particle/cell entrapment, FDTD

## Abstract

A gold-coated array of flow-through inverse pyramids applicable as substrate for entrapment and immobilization of micro-objects and for surface enhanced Raman spectroscopic measurements was fabricated using bulk micromachining techniques from silicon. Surface morphology, optical reflectance, immobilization properties, and surface enhanced Raman amplification of the array were modelled and characterized. It was found that the special perforated periodic 3D structure can be used for parallel particle and cell trapping and highly sensitive molecular analysis of the immobilized objects.

## 1. Introduction

Being a non-contact, fast, and relatively easy material characterization technique requiring no sample preparation, Raman spectroscopy is finding many applications in biology, life sciences, and other areas. However, Raman scattering is inherently weak (the Raman cross-section can be 10^12^–10^14^ times smaller than that of fluorescence [1]), which prohibits its use for the detection of analytes having low concentration. Nevertheless, Raman sensitivity can be improved by implementing surface-enhanced Raman scattering (SERS) [2], where the degree of achievable sensitivity can reach up to attomolar (10^−18^ M) concentrations, as has been observed in many cases [3,4]. As a spectroscopic tool, SERS has the potential to combine the sensitivity of fluorescence with the structural information given by Raman spectroscopy.

During SERS the scattering takes place in close vicinity of the nanostructured metallic surfaces (nanoparticles or solids having nanoscopic morphology [5]). The interaction of the electromagnetic field of photons with the surface plasmons of the metallic nanostructure produces an enhancement of the Raman signal; the gain can be several orders of magnitude. The key factor to achieve a successful SERS experiment is to find an appropriate SERS-active agent (nanoparticles or substrate) that will provide the required efficiency at a given excitation wavelength and to localize the analyte to be measured in close vicinity of this agent.

Metallic (mostly gold, silver, and copper) nanoparticles of different sizes and geometries [6,7], porous structures [8], as well as periodically patterned surfaces [9,10] were found to be efficient SERS-active materials [5]. Periodic arrays of inverse pyramids with characteristic size of a few micrometers were among the first SERS active substrates finding commercial utilization [11]. Other periodic surfaces, such as inverse hemispheres and rounded pyramids, showed good performance as well [9]. However, patterned substrates consisting of an array of closed voids lack the appropriate flow characteristics required for proper localization of the analytes near the SERS-active surface, and this affects their efficiency. This can be avoided by designing structures with flow-through channels within the voids that will promote movement of the analytes to the surface. In addition, specific geometries can be fabricated to entrap micro-objects such as blood cells.

In this work a special SERS-active substrate was prepared for flow-through applications, particle and cell entrapment, and highly sensitive detection of molecules immobilized on the surface. The highly efficient inverse pyramid array was modified to be able to serve as an ensemble of cavities in a perforated membrane applicable for particle and cell filtering, sorting, trapping, and subsequent SERS characterization. To demonstrate our concept, different polymer microparticles were trapped in the voids of the gold coated perforated substrate and their SERS signals were detected.

## 2. Materials and Methods

**Figure 1:**
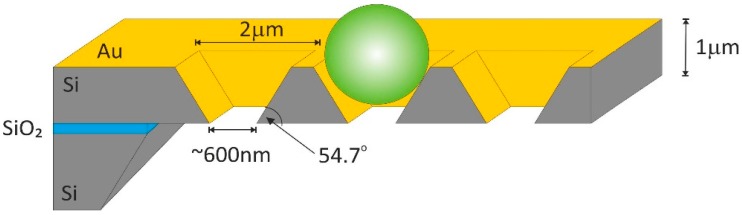
Schematic representation of the flow-through surface-enhanced Raman scattering (SERS) substrate with an entrapped microsphere.

The array of periodic perforated inverse pyramids was prepared on a polished silicon-on-insulator (SOI) wafer. The 3D structure was shaped, using an appropriate masking pattern, by alkaline etching from the front side in the 1 µm thick device layer of the wafer. Based on the sizes of commercially available closed inverse pyramid arrays, the appropriate size of the initial pattern was calculated to develop adequate trap geometry defined by the <111> planes of the silicon crystal. Periodic vertically tapered microchannels were formed by etching away the back-side handler silicon and buried oxide. The resulting structure was coated with a 150 nm thick evaporated layer of gold. The obtained geometry (Figure 1) was applied as an array of particle traps and SERS substrate.

Scanning electron microscopy was performed using a Zeiss LEO 1540XB (Carl Zeiss, Jena, Germany) crossbeam field emission scanning electron microscope (FESEM). Optical micro-reflection was measured on an Olympus BX51 (Olympus, Hamburg, Germany) microscope combined with a Princeton Instruments (PI) Isoplane SCT320 imaging spectrograph and a PI PIXIS:400BRX CCD detector. Raman spectra were recorded on a Renishaw 1000 micro-Raman spectrometer attached to a Leica DM/LM microscope. A 785 nm diode laser was used as an excitation source and it was focused into a spot of 1 µm diameter on the sample. A benzene derivative (benzophenone) dissolved in isopropyl alcohol with concentration of 1 mmol was used to test the SERS enhancement of the structure. The solution was dripped onto the array and the measurements were carried out after the evaporation of the solvent. Two types of fluorescent polystyrene latex microbeads were used for the demonstration of particle entrapment and for mapping Raman measurements. The beads were purchased from Sigma Aldrich (Sigma Aldrich, St. Louis, MO, USA) (SA sample) and Spherotech (ST sample) and their physical properties are summarized in Table 1. The fluorescent imaging of the trapped microbeads was performed by a Zeiss AxioVert A1 fluorescent microscope using its 38 GFP/FITC (λ_ex._/λ_em._: 470 nm/525 nm) and 49 DAPI (λ_ex._/λ_em._: 365 nm/445 nm) filter sets, respectively.

The near-field intensity distributions of perforated inverse pyramid arrays were studied by finite-difference time-domain (FDTD) simulations using the Lumerical FDTD Solutions v.8.15.736 software. Perfectly matched layer (PML) boundary conditions were chosen to model the periodic array geometry. The size of the simulation cell was set according to the geometry of the fabricated array structure to 2.9 × 2.9 × 4.6 µm. Silicon was selected as the substrate material with a 150 nm gold coating on top of it. The software’s built-in material parameter set from CRC Handbook of Chemistry and Physics [14] was used for the gold layer and that from the Handbook of Optical Constants of Solids I [15] for the silicon substrate. The simulation grid was defined by the built-in auto-mesh algorithm with an accuracy level of 4. The inverse pyramid array was illuminated using a broadband (400–900 nm) plane wave having normal incidence to the array surface and polarization parallel to one of the bases of the pyramid. Near-field profiles were recorded using two monitors placed along the two symmetry planes of the pyramid, being perpendicular to the array surface, and a third one placed above the surface.

## 3. Results and Discussion

**Figure 2:**
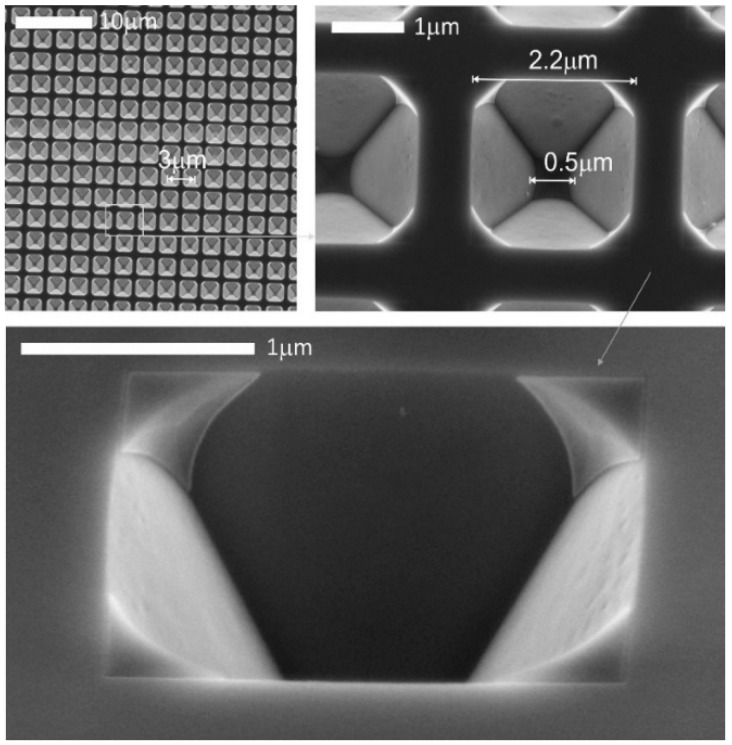
Scanning electron microscopy (SEM) image of the periodic perforated SERS substrate with dimensions of the voids.

Figure 2 shows SEM images of the developed SERS substrates. It can be seen that a regular periodic array of etched structures was created on the silicon surface. The perforated inverse pyramids have a final base size of 2.2 × 2.2 µm (corresponding to 2.5 × 2.5 µm size of the silicon structure without the 150 nm gold coating) due to the lateral underetching. The isotropic etching resulted in flat lateral faces of the pyramids with sharp edges. A 0.5 × 0.5 µm bottom opening was created at the center of the inverse pyramid allowing the flow-through of the sample and trapping of the objects having dimensions greater than the channel sizes of the inverse structure.

**Figure 3:**
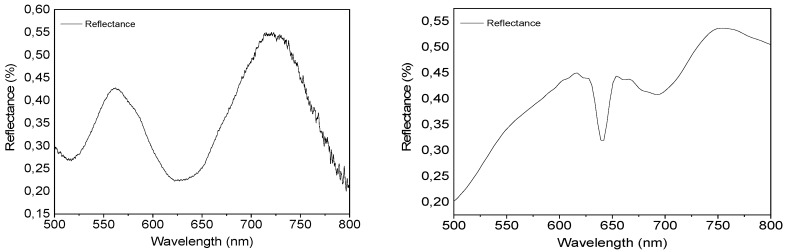
Experimentally measured (**left**) and finite-difference time-domain (FDTD) simulated (**right**) reflectance spectrum of the inverted pyramids.

Micro-reflectance of a single inverted pyramid structure was measured in the 500–800 nm region. Light reflected from an unstructured gold surface of the same substrate was used as the reference. The data are provided in Figure 3. The periodic structure shows the lowest reflectance in the 620–660 nm wavelength region and above 780 nm. The low reflectance is related to local plasmon resonance features and, thus, to the surface enhancement properties of the gold coated periodic array. Data in Figure 3 indicate that this structure should have high plasmonic enhancement in the abovementioned regions.

The reflectance spectrum obtained from the FDTD simulations is provided in Figure 3 as well. In general, the reflectance increases toward higher wavelengths, however, several minima can be observed in the 620–750 nm region. The reflectance sharply decreases from 0.3 to 0.2 between 630 and 650 nm with the minimum located at 642 nm. There is another broader region of decrease between 660 and 750 nm with local minima at 675 and 690 nm. The difference between the simulated and experimental spectra can be attributed to the contribution from the flat mid-pyramid regions to the latter, the reflection spectrum of which should be very similar to that of the reference flat gold surface. In spite of the differences, both spectra indicate that the plasmonic behavior is expected to appear in the 600–800 nm region.

**Figure 4:**
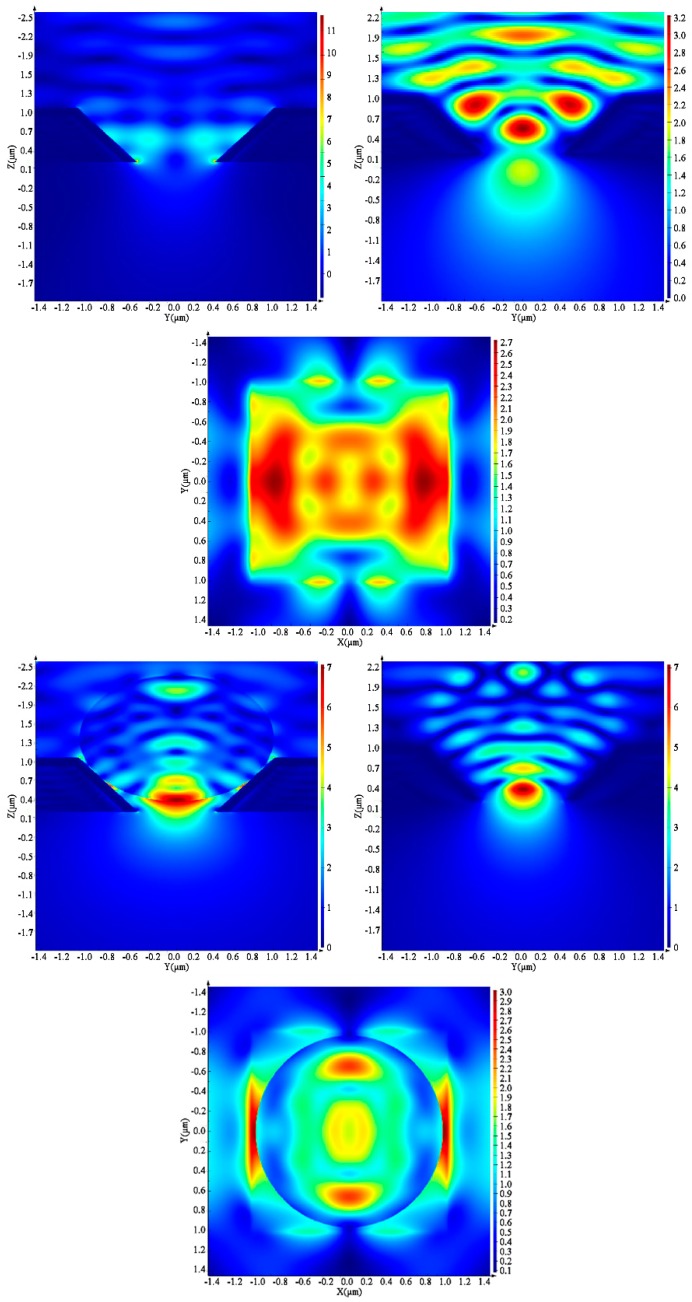
Simulated near-field intensity distribution of gold-coated perforated inverse pyramid without (upper row) and with (lower row) a trapped polymer microparticle in different planes.

The near-field intensity distribution developing in the 3D SERS substrate was simulated to analyze the molecule recognition capability of the architecture. The inverted pyramid array structure was modelled without and with a trapped microsphere as demonstrated in Figure 4. The simulated near-field intensities are shown for the three different planes described earlier. For the structure without a microparticle, the hotspots are mainly distributed along the symmetry plane of the pyramid being parallel with the polarization of the incident light, with maximum enhancement factor of 3.2. However, the highest enhancement of 12.0 units can be seen in the other plane, at the bottom edge of the truncated pyramid. Other local maxima can be seen in the surface plane of the structure, close to the edges of the pyramid.

The near-field distribution changes remarkably after the addition of the polymer microparticle to the model. Significant enhancement can be observed near the contact points of the sphere and the sides of the pyramid (with maximum enhancement factors of 3.0), prognosticating an effective surface enhanced Raman spectroscopic analytical capability for molecules situated close to the bead surface or immobilized on it. The hotspot with highest enhancement, however, is around the particle surface close to the bottom opening, where a factor of 7.0 can be observed.

**Figure 5:**
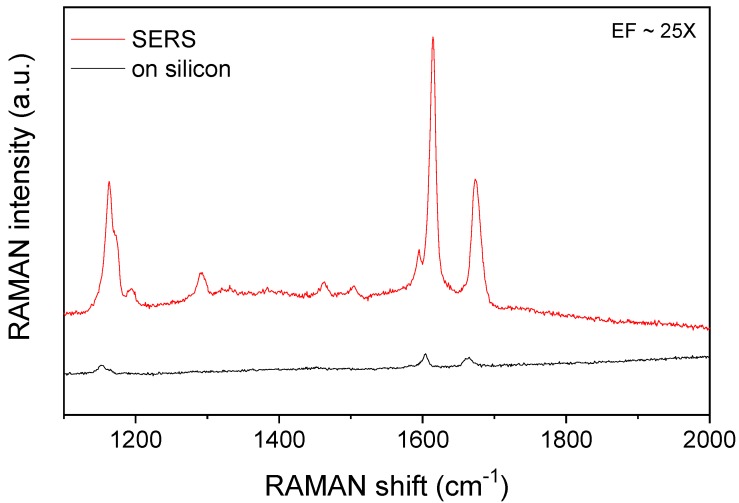
Comparison of the Raman signals of benzophenone detected on silicon, and on the periodic array of perforated pyramidal structures.

The SERS enhancement of the 3D periodic structure was tested with the dripping method, by comparing the Raman spectra of benzophenone recorded on flat silicon and on the structured gold surface, respectively. The two spectra are shown in Figure 5. The characteristic bands of benzophenone appear in both spectra but with remarkably different intensities. The enhancement factor, determined from the intensity ratio of the SERS and normal Raman peaks was found to be 25, calculated for the 1603 cm^−1^ band. This value is not too high; however, the measurement conditions are different. Measurements performed earlier with the benzophenone on an array of closed voids of the same size showed enhancement factors of similar scale [16]. While the entire excitation spot covers the sample on the silicon surface, in the other case a part of it falls on the bottom opening of the pyramid so the Raman scattering is excited on a much smaller area. In addition, the concentration of the analyte on the horizontal reference surface and on the tilted faces of the perforated inverse pyramids is also different. While all the benzophenone is drying on the flat surface, most of the solution flows through the bottom opening of the perforated voids. Nevertheless, the observed Raman gain indicates the SERS activity of the array.

**Figure 6:**
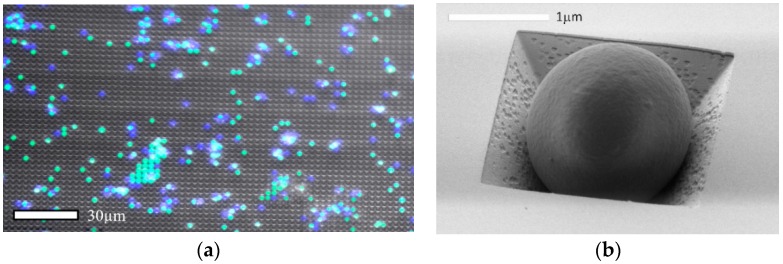
Fluorescent beads with approximately 2 µm diameter (SA—green and ST—blue) entrapped in the periodic array of perforated pyramidal structures: multichannel fluorescent image (**a**) and SEM image (**b**).

In order to test the performance of the flow-through array as a particle trap and as a SERS enhancement surface, polystyrene microparticles labelled with different fluorescent molecules were mixed with water and dripped onto the periodic structure. The particles having sizes (2 µm diameter) comparable to the periodic 3D structures were entrapped on the surface after washing. The lateral distribution of the beads of different emission wavelength (SA: green at cca. 505 nm and ST: blue at cca. 445 nm) determined by fluorescent microscopy is shown in Figure 6a. It can be seen that several microparticles were immobilized on the flow-through substrate and it is easy to distinguish them by fluorescence microscopy. The SEM image also shows an entrapped microbead in Figure 6b.

**Figure 7:**
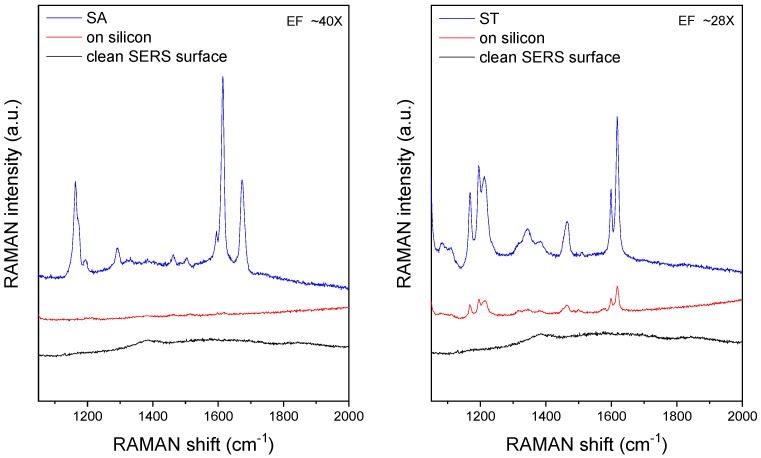
Comparison of SERS spectra recorded on pristine SERS substrate, as well as of different fluorescent beads on silicon surface and entrapped in the periodic array.

Bonding configuration of the polystyrene beads was analyzed by SERS utilizing the signal enhancement of the structured surface of the traps. The definite and sensitive differentiation of the structures is presented in Figure 7. A total of three spectra were recorded for each type of beads: one on the clean array surface, one on a microparticle placed on a flat silicon surface, and one on a bead immobilized in the array. No Raman bands were observed in the spectra measured on the clear gold-coated array; only some low-intensity broad bands were detectable between 1400–2000 cm^−1^, presumably representing a weak fluorescence background due to contaminants adsorbed on the surface. The characteristic Raman bands of the beads are of very low intensity in the spectrum of the SA sample recorded on silicon; these are more intense in the case of the ST microbead. Raman peaks are of much higher intensity and are easily detectable in the spectra of the beads entrapped in the flow-through SERS array. The SERS enhancement factors determined from intensity ratios were found to be 40 (for SA) and 28 (for ST). These values are affected by the remarkable non-SERS contribution, being present in the SERS spectrum as well, arising from the volume of the microbead being far from the hotspots. Therefore, the real enhancement values should be much higher. Additional tests are planned with a molecule forming a single monolayer on the gold and polymer surface in order to determine the absolute SERS enhancement ratio of the flow-through substrate.

The peaks observable in the SA and ST samples on gold and silicon surfaces are summarized in Table 2. For reference, characteristic Raman bands of polystyrene are also provided. Only some of the polystyrene peaks can be detected in the spectrum of the SA sample on silicon, while, in addition to the PS peaks, new bands appear in the SERS spectrum of the SA sample at 1292, 1505, and 1674 cm^−1^. These correspond to the fluorescein molecules and carboxyl group. The recorded spectra clearly show the presence of fluorophore molecules near the surface of the ST spheres as well. Here, the PS and some other bands can be detected in both normal and SERS spectrum of the ST sample, but there are new peaks appearing in the SERS spectrum at 1084 and 1110 cm^−1^.

**Figure 8:**
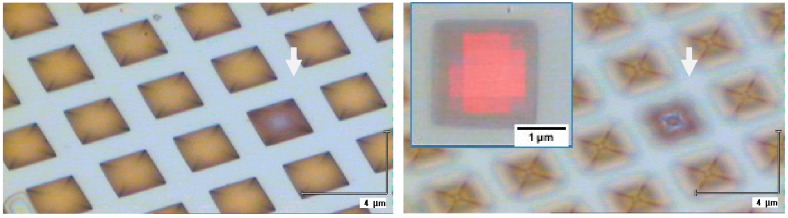
Optical microscopic images recorded with microscope focused on the top and the bottom of the inverse pyramid SERS array. The arrow shows the location of the microbead in one of the voids. The insert shows the Raman map as an overlay.

Mapping Raman measurement was also performed on one of the entrapped microbeads. Figure 8 shows series of optical microscopic images recorded on the region of the substrate used for this measurement. Because of the small depth of focus it is difficult to recognize the entrapped microparticle placed on the inverse pyramid (marked with an arrow), but it can be seen on the image that the mapped void contains an object. The Raman intensity map recorded over the entrapped microbead is shown in Figure 8 as well. The intensity distribution of one of the Raman peaks of the microbead over the array unit entrapping it reveals the spherical shape of the particle.

## 4. Conclusions

A perforated periodic 3D structure consisting of gold coated truncated inverse pyramids was designed and fabricated that can be used for particle (or cell) trapping and extreme sensitive detection of molecules situated near or immobilized on the surface of the confined beads using surface-enhanced Raman spectroscopy. The voids are 1 micrometer thick, with the base size of 2.2 × 2.2 micrometers on the top surface and a 0.5 × 0.5 micrometer opening on the bottom. It was found that the periodic array can efficiently trap and immobilize microbeads of appropriate size. The near-field enhancement properties were modelled with FDTD simulations and demonstrated experimentally with surface enhanced Raman spectroscopic measurements using near infrared excitation. In addition, the immobilized objects were recognized using fluorescent microscopy as well.

## Figures and Tables

**Table 1:** Physical properties of the applied fluorescent microbeads.

Name	Supplier	Diameter	Core Material	Refractive Index	Exc./Em. Wavelength	Surface Modification
SA	Sigma Aldrich	2.0 µm ± 0.2 µm	polystyrene latex	1.612 [12] (bulk: 1.5916 [13])	470 nm/505 nm	carboxilated
ST	Spherotech	1.970 µm ± 0.049 µm	polystyrene	1.612 [12] (bulk: 1.5916 [13])	395 nm/445 nm	n.a.

**Table 2:** Peak positions in normal Raman and SERS spectra of Sigma Aldrich (SA) and Spherotech (ST) samples.

Raman Peak Position, cm^−1^				
*SA Sample*		*ST Sample*		*Polystyrene*
SERS	Normal Raman	SERS	Normal Raman	Normal Raman
		1110		
1163		1168	1168	1156
1194		1194	1195	1196
		1212	1212	
1292				
1333		1343	1343	1326
		1383	1383	
1463		1465	1465	1448
1505				
1595		1599	1599	1600
1614	1614	1618	1618	
1674				
